# Socio-demographic determinants of childhood immunization incompletion in Koforidua, Ghana

**DOI:** 10.1186/s13104-018-3767-x

**Published:** 2018-09-10

**Authors:** Reindolf Anokye, Enoch Acheampong, Amy Budu-Ainooson, Anthony Kwaku Edusei, Paul Okyere, Joslin Dogbe, Alberta Nadutey

**Affiliations:** 1 Centre for Disability and Rehabilitation Studies, Department of Community Health, Kwame Nkrumah University of Science and Technology, Kumasi, Ghana; 2 Department of Health Education and Promotion, School of Public Health, Kwame Nkrumah University of Science and Technology, Kumasi, Ghana

**Keywords:** Socio-demographic factors, Incompletion, Childhood immunization, Mothers, Children under 5 years

## Abstract

**Objective:**

Immunization saves more than 3 million lives worldwide each year, and it saves millions from suffering illness and lifelong disability. The study sought to assess the socio-demographic factors that influence childhood immunization incompletion. A cross-sectional descriptive design was employed for the study conducted at the Child Welfare Clinic in the Regional Hospital, Koforidua. A total of 280 caregivers/mothers who have children aged between 0 and 59 months were included in this study. Data were entered and analyzed using SPSS.

**Results:**

The study found that being divorced (*p* = 0.048) and working part-time (*p* = 0.049) has a significant and positive association with immunization incompletion. Women who were divorced [AOR (95% CI) 3.01 (1.59–58.2)] were 3 times less likely to complete immunization than those who were cohabiting, married and widowed taken into account the effect due to all the additional confounder variables included in the analysis. Women who were working part-time were 2.28 times less likely to complete immunization schedule than those working full-time; [AOR (95% CI) 2.28 (1.031–9.11)]. This study has documented socio-demographic factors influencing childhood immunization incompletion in the Regional Hospital, Koforidua. The Ministry of Health should, therefore, put in measures like public education to encourage mothers to complete each immunization schedule.

**Electronic supplementary material:**

The online version of this article (10.1186/s13104-018-3767-x) contains supplementary material, which is available to authorized users.

## Introduction

Childhood immunization has been established as one of the key interventions in Public Health that has resulted in child morbidity reduction [1]. It is therefore important that a child receives the complete recommended immunization dosage at the right age in order to escape childhood diseases that are vaccine-preventable [1].

The incidence of disabilities, including mental retardation as well as mobility impairment, has been greatly reduced by immunization against polio as well as rubella, meningitis, and measles [2, 3]. Vaccines have contributed to the reduction of intellectual disability caused by measles encephalitis, congenital rubella syndrome, and Haemophilus influenza type b meningitis or Hib [4].

According to the Centers for Disease Control and Prevention (CDC), brain damage may occur when a child catches pertussis (whooping cough), measles, mumps, Hib disease, or varicella (chicken pox). Encephalitis, an inflammation of the brain, is the cause of brain damage in pertussis, measles, and mumps which can be prevented through immunization [5, 6].

Immunization has brought sound health to many children in the world [7, 8] and have been effective in protecting children against vaccine-preventable diseases in low and middle-income countries [9, 10].

Presently, it is recommended that at birth all children should be vaccinated with Bacille Calmette–Guerin (BCG) as well as a total of four doses of oral polio vaccine (OPV). By the 6th, 10th and 14th week, all children should be vaccinated with OPV 0, OPV1, OPV 2 and OPV 3 respectively. A further three doses of diphtheria, pertussis, tetanus, Haemophilus influenza type B and Hepatitis B (DPT/HiB/HepB) 5-in-1 vaccine should be given on the 6th, 10th and 14th week respectively. On the 9th month, each surviving child should be given Measles and Yellow fever vaccines [11, 12]. Therefore, one can conclude that, if a child misses at least a dose out of the eight vaccines recommended when he/she is under 12 months old, that child has not completed the immunization schedule [11, 12].

This study, therefore, assesses the socio-demographic determinants of Childhood Immunization Incompletion in Koforidua, Ghana.

## Main text

### Methods

This study was conducted at the Child Welfare Clinic in the Regional Hospital, Koforidua in the New Juaben district of Eastern Region. The Koforidua Regional Hospital serves as a referral specialist’s center that offers specialist medical and surgical care. The hospital also trains interns and residents as well as allied health students. A clinic setting was selected because the investigators wanted to select mothers who had started the immunization already but have missed some schedules due to some socio-demographic factors.

For this study, a retrospective descriptive cross-sectional design was adopted using a quantitative approach. In this case, the design was adopted to describe the socio-demographic factors that influence childhood immunization incompletion. Mothers were selected for the study using a simple random sampling technique. They were asked to pick pieces of papers that were folded with ‘Yes’ and ‘No’ written in it. The mothers who picked ‘Yes’ were selected until the determined sample size was achieved. The target population considered eligible for this study were all mothers and caregivers at the Child Welfare Clinic (CWC) at the regional hospital, Koforidua. For the period of data collection, 800 mothers were found at the study site. Using the formula of Yamane [13] for determining samples, 95% confidence level and a precision of 0.05 were used to calculate for the sample using the Equation;

$$n = \frac{N}{{1 + N(e)^{2} }}$$

*n* represents the sample size, *N* represents the population size, and *e* represents the level of precision.

$$N = 800$$

$$1 + N (e)^{2} = { 1 } + { 8}00 \, \left( {.0 5} \right)^{ 2}$$

$$n = \frac{800}{{1 + 800 (.05)^{2} }}$$

$$n = 267$$

Since the total population was 800 and 267 was selected as a sample size, the margin of error was 4.9. Using the 95% confidence level and a level of precision of 0.5, it is claimed therefore that there is a 95% chance that the confidence interval that was calculated contains the true population mean with a margin of error of 5%. The 5% margin of error implies that even if all 800 mothers are used in this study, the results may be different if we subtract 5% or add 5% to the original score. Assuming that 5% of the sample will not be willing to be part of the study, 13 (representing 5% of the achieved sample of 267) were added to 267 to give us a sample size of 280. The number for the non-respondents rate was selected because it was expected that some respondents will opt out at a certain stage or will not be willing to be part of the study.

In collecting data from the respondents, a structured questionnaire was used as the data collection instrument. Questionnaires were administered to respondents that consented to participate in the study. The questionnaire comprised of respondents demographic data and questions to guide the researchers in describing factors that influence childhood immunization incompletion (Additional file 1). Data was sought from Hospital records and vaccination cards, as to the immunization status of the children to confirm what respondents stated. The questionnaires were directly administered to respondents by the researcher and some selected research assistants. Data were collected weekly for 4 weeks to increase the probability of having varied opinions.

The results obtained were analyzed using the Statistical Package for the Social Sciences (SPSS) version 22. Prior to this, data was dissected to ensure all possible errors are removed by all the investigators. The research results were presented in tables as this was to help present the results of the work in a graphical and appropriate manner for easy interpretation and presentation. Permission was sought from the hospital management and the in-charge of the units under which the research was conducted. Participants were assured that the information they provided was strictly going to be confidential. Information generated was strictly used for academic purposes of which was stored appropriately at a place determined by the School.

### Results

#### Socio-demographic characteristics of respondents

Table 1 indicates the demographic characteristics of respondents who participated in the study. From the table, the highest age group was 24–30 years representing 22.8%. The majority (67.1%) of the participants were married and had tertiary education whiles 40% had children between 3 and 4 years. Also, close to half (40%) of the respondents were employed as full-time workers. Most (28.6%) of the respondents received income between 100 and 200 GH cedis and the most common religion was Christianity (64.2%).

**Table 1:** Socio-demographic characteristics of respondents

Variable	Frequency (n = 280)	Percentage
Age (years)		
13–17	4	1.4
18–23	64	22.8
24–30	116	41.4
Above 30	96	34.2
	Mean = 21.3	SD = 2.12
Age of child		
Less than 1 year	86	31
1–2 years	82	29
3–4 years	112	40
	Mean = 2.09	SD = 1.11
Marital status		
Single	44	15.7
Married	188	67.1
Divorce	12	4.3
Widow	4	1.4
Cohabiting	32	11.4
Level of education		
Primary	16	5.7
JHS	32	11.4
Secondary	76	27.1
Tertiary	152	54.3
No formal education	4	1.6
Employment status		
Unemployed	84	30.0
Working part-time	84	30.0
Working full-time	112	40.0
Level of income		
< 100	68	24.3
100–200	80	28.6
200–300	56	20.0
Above 300	76	27.1
	Mean = 180	SD = 1.43
Religion		
Christian	180	64.2
Muslim	96	34.3
Traditionalist	4	1.4

#### The relationship between socio-demographic factors and immunization completion

Table 2 shows the relationship between socio-demographic factors and immunization completion. From the table, there were significant differences in immunization schedule completion among women with different marital status (*p* = 0.041) as well as different employment status (*p *= 0.013). The immunization schedules before and after 6 months can be found in Additional file 2: Table S1 and Additional file 3: Table S2.

**Table 2:** Showing the relationship between socio-demographic factors and immunization completion

Variable	Completed immunization		Did not complete immunization		Total	
	Frequency	Percentage	Frequency	Percentage	Frequency	Percentage (%)
Mother’s age						
13–17 years	0	0.0%	4	100.0%	4	100.0
18–23 years	28	43.8%	36	56.3%	64	100.0
24–30 years	48	41.4%	68	58.6%	116	100.0
Above 30 years	48	50.0%	48	50.0%	96	100.0
Total	124	44.3%	156	55.7%	280	100.0
	Chi Square = 1.214		df = 3	p = 0.750		
Age of child						
Less than 1 year	29	41%	41	59%	70	100
1–2 years	46	42%	66	58%	113	100
3–4 years	49	50%	49	50%	98	100
Total	124	44%	156	56%	280	100
	Chi Square = 1.004		df = 2	p = 0.051		
Marital status						
Single	20	45.5%	24	54.5%	44	100.0
Married	100	53.2%	88	46.8%	188	100.0
Divorce	0	0.0%	12	100.0%	12	100.0
Widow	0	0.0%	4	100.0%	4	100.0
Cohabiting	4	12.5%	28	87.5%	32	100.0
Total	124	44.3%	156	55.7%	280	100.0
	Chi Square = 7.981		df = 3	p = 0.041		
Level of education						
Primary	13	75.0%	5	25.0%	18	100.0
JHS	33	42.1%	45	57.9%	78	100.0
Secondary	37	32.1%	77	67.9%	114	100.0
Tertiary	37	52.9%	33	47.1%	70	100.0
Total	120	42.6%	160	57.4%	280	100.0
	Chi Square = 3.714		df = 2	p = 0.294		
Employment status						
Unemployed	44	52.4%	40	47.6%	84	100.0
Working part-time	12	14.3%	72	85.7%	84	100.0
Working full-time	68	60.7%	44	39.3%	112	100.0
Total	124	44.3%	156	55.7%	280	100.0
	Chi Square = 11.281		df = 3	p = 0.013		
Mother’s income						
< 100	9	52.9%	8	47.1%	17	100.0
100–200	9	45.0%	11	55.0%	20	100.0
200–300	4	28.6%	10	71.4%	14	100.0
Above 300	8	44.4%	10	55.6%	18	100.0
Total	30	43.5%	39	56.5%	69	100.0
	Chi Square = 1.911		df = 3	p = 0.591		
Religion						
Christian	19	42.2%	26	57.8%	45	100.0
Muslim	11	45.8%	13	54.2%	24	100.0
Traditionalist	1	100.0%	0	0.0%	1	100.0
Total	31	44.3%	39	55.7%	70	100.0
	Chi Square = 1.359		df = 2	p = 0.507		

Odds ratio with 95% confidence interval was further used to determine the association between socio-demographic factors and immunization completion as shown in Table 3. From the table, women who were divorced [AOR (95% CI) 3.01 (1.59–58.2)] were 3 times less likely to complete immunization than those who were cohabiting, married and widowed taken into account the effect due to all the additional confounder variables included in the analysis. Women who were working part-time were 2.28 times less likely to complete immunization schedule than those working full-time; [AOR (95% CI) 2.28 (1.031–9.11)].

**Table 3:** Odds ratio with 95% confidence interval for association between socio-demographic factors and immunization completion

Variable	Completed immunization		Univariate		Multivariate^a^	
	Yes	No	OR (95% CI)	p-value	AOR (95% CI)	p-value
Mother’s age (years)						
13–17	0	4	1.00		1.00	
18–23	28	36	0.49 (0.41–3.02)	0.797	1.21 (0.35–4.31)	0.122
24–30	48	68	2.66 (1.13–20.28)	0.121	1.38 (0.04–2.29)	0.211
Above 30	48	48	3.12 (1.98–21.0)	1.432	1.99 (0.12–2.01)	0.912
Age of child						
Less than 1 year	29	41	1.00		1.00	
1–2 years	46	66	3.42 (2.91–3.91)	1.26	2.98 (1.92–3.04)	0.093
3–4 years	49	49	4.23 (3.11–4.87)	0.09	3.12 (2.01–3.92)	0.122
Marital status						
Single	20	24	1.00		1.00	
Married	100	88	2.01 (1.32–3.33)	0.139	1.21 (0.28–3.27)	0.503
Divorce	0	12	12.5 (3.54–45.49)	0.034	3.01 (1.59–58.2)	0.048
Widow	0	4	1.21 (0.21–3.21)	1.09	1.34 (0.72 –1.92)	0.122
Cohabiting	4	28	6.33 (0.08–1.56)	0.112	0.48 (0.08–2.46)	0.210
Level of education						
Primary	13	5	1.00		1.00	
JHS	33	45	1.02 (0.44–2.35)	0.838	1.62 (0.41–4.60)	0.425
Secondary	37	77	1.14 (0.32–4.01)	0.912	2.00 (0.17–21.05)	0.291
Tertiary	37	33	1.39 (0.81–1.91)	1.109	1.09 (0.14–1.78)	0.431
Employment status						
Unemployed	44	40	1.00		1.00	
Working part-time	12	72	8.21 (3.12–20.18)	0.02	2.28 (1.031–9.11)	0.049
Working full-time	68	44	3.02 (2.81–2.97)	1.100	2.10 (1.082–1.93)	1.129
Mother’s income						
< 100	9	8	1.00		1.00	
100–200	9	11	3.09 (1.98–2.96)	0.762	2.41 (1.56–2.01)	0.431
200–300	4	10	1.10 (0.48–2.48)	0.797	0.81 (0.14–3.39)	0.690
Above 300	8	10	1.45 (0.65–0.99)	1.07	1.73 (0.76–1.01)	0.07
Religion						
Christian	19	26	1.00		1.00	
Muslim	11	13	3.11 (1.10–12.11)	0.912	0.22 (0.18–2.95)	0.218
Traditionalist	1	0	1.89 (0.21–1.02)	1.321	2.12 (1.82–2.03)	0.982

*OR* odds ratio, *CI* confidence interval, *AOR* adjusted odds ratio

^a^Mutually adjusted

### Discussion

The highest age range of 24–30 years found in this study was consistent with the study conducted by Mustafi and Azad [14]. This implies that most mothers in both study locations may usually fall within the ages of 24–30 years. In terms of education, more than half of the respondents (54.3%) had a higher education which contrasted with the study conducted by Abdulraheem et al. [15]. The difference could be as a result of the study locations as this present study was conducted in an urban setting in Ghana whereas the earlier study was conducted in rural Nigeria.

Maternal age was not a predictor of immunization incompletion in this study. The finding is inconsistent with other studies where maternal age was a predictor of vaccination incompletion [16]. However, a cross-sectional study done in Mozambique revealed that mothers’ age had no significant relationship with respect to immunization incompletion [17]. In other settings, both younger [18] and older age of mothers [19] has been reported to be associated with incomplete vaccination. The probable reason for the observed discrepancy may be due to the differences in socio-cultural characteristics of the two-study populations and exposure to immunization information during and after pregnancy.

Although other factors were key barriers to immunization incompletion, the findings of this study showed no significant association between education and immunization incompletion. This finding was consistent with findings of Ethiopia DHS data of 2005 which revealed no association between caretakers educational level and vaccination incompletion [20]. Nonetheless, previous studies by Marks et al. [21] revealed that the educational status of mothers has a strong association with a high vaccine uptake. Also, a study by Abdulraheem et al. [15] confirms this assertion from Marks et al. [21] that educational level is associated with mothers and missed opportunities for vaccination. This implies that education cannot be overlooked when assessing factors influencing childhood immunization incompletion.

The study showed that religion of respondents has no significant association with child vaccination incompletion which is in contrast with other study findings by Kulig et al. [22] among others. Some studies revealed that some religious bodies are known to discourage their members from accepting vaccination [22, 23]. Also, Religion played a role in the risk of non-immunization in a Nigerian study where a significantly higher proportion of non-immunized children were observed among mothers who were Muslim. Children with partial immunization were also found to be significantly higher among Muslim whiles full/completed immunization was observed at a significantly higher level among mothers who were Christian [24]. Nevertheless, a study in Mozambique was consistent with this study which showed no significant association between religion and vaccination incompletion. Differences in results obtained by different investigators who looked at the influence of religion on vaccination may be due to differences in socio-cultural antecedents and theological persuasions between populations involved in the studies.

In this study, significant relationships were found between marital status and immunization incompletion. A study by Falagas and Zarkadoulia [25], also agrees with this finding. Conversely, other studies have shown a no significant influence of marital status on immunization completion [16, 17]. This implies that the marital status of the mothers might influence their decision to go to the health centers to complete the immunization schedule of the child.

Furthermore, the employment status of the respondents was found to have a significant association with immunization incompletion. This is in line with a study conducted by Omole and Owodunni [26] and Abdulraheem et al. [15], which showed that income and employment status have a significant relationship with immunization completion. Antai [24], found that being employed was significantly associated with a higher likelihood of the child being fully immunized. The reason for this outcome might come from the fact that the days of immunization might impede the mother’s employment schedule.

Several studies have found a relationship between wealth status and vaccination status [27, 28]. Children from wealthy households may be more likely to have their vaccination status checked and to receive missing doses of vaccines when attending a healthcare facility than children from poor households. The findings of this study found no significant positive relationship between the income level of mothers and immunization incompletion contrary to earlier studies. This could be attributed to the fact that access to a health facility in the area is not limited by income probably due to the introduction of a National Health Insurance Scheme that covers a certain aspect of the cost incurred by mothers when they visit a healthcare facility in Ghana.

### Conclusion

This study has documented socio-demographic factors influencing childhood immunization incompletion in the Regional Hospital, Koforidua. Determinants of receipt of complete and incomplete immunization are complex and interwoven. This study ascertained several socio-demographic factors influencing childhood immunization incompletion, key among them were socio-demographic factors such as being divorced and working part-time.

## Limitations

The study had some limitations which included recall bias where the mother might forget the vaccination/immunization status of their children and misclassification. Also, cultural factors were not studied during this analysis; a more in-depth look at various practices of parenting might illuminate ways it influenced their behavior. However, the findings were not compromised in any way.

## Additional files

**Additional file 1.** Questionnaire.

**Additional file 2: Table S1.** Immunization schedules before Six (6) months.

**Additional file 3: Table S2.** Immunization schedule after Six (6) months.

## Abbreviations

BCG: Bacille Calmette–Guerin
DPT: diphtheria, pertussis, tetanus
OPV: oral polio vaccine
SPSS: Statistical Package for Social Sciences
