# Supplementary material for: Socio-demographic determinants of childhood immunization incompletion in Koforidua, Ghana
Source: BMC Res Notes. 2018 Sep 10;11:656. doi: 10.1186/s13104-018-3767-x (PMC6131842; doi:10.1186/s13104-018-3767-x)
Supplement: Supplementary file 1 — Additional file 1. Questionnaire. [file 13104_2018_3767_MOESM1_ESM.docx]

**Additional file 1**

**QUESTIONNAIRE**

**Participant ID**: ………………….. **Date**: ………………………

**INSTRUCTIONS:** ***Please tick (√) the right answer(s)***

**SOCIO – DEMOGRAPHIC CHARACTERISTICS**

1. Age of mother
2. 13 – 17 years b. 18 – 23 years c. 24 – 30 years
3. Above 30 years

2. Age of Child …………………

3. What is your marital status?

1. Single b. Married c. Divorce
2. Widow e. Cohabiting

4. What is your religion?

1. Christian b. Muslim c. Traditionalist
2. Others …………….

5. What is your Education Level?

1. Primary b. JHS c. Secondary
2. Tertiary e. Not Educated

6. What is your current employment status?

1. Unemployed b. Working part-time c. Working full –time
2. Retired

7. What is your monthly income (GHS)?

1. < 100 b. 100 – 200 c. 200 – 300
2. Above 300

**IMMUNIZATION SCHEDULE**

8. What was your child given at birth?

1. BCG/OPV b. BCG c. OPV
2. Measles e. I don’t know f. Not given any
3. Was your child given Polio, DPT/Hep B/Hep 1(5 in 1), Pneumococcal vaccine, Rotavirus at six (6) weeks?
4. Yes b. I don’t know
5. No, why ……………………………………….
6. At 10 weeks, what did your child receive, multiple choices
7. Polio b. DPT/Hep B/ Hep1 (5 in 1) c. Rotavirus
8. Pneumococcal vaccine e. None, why……………………………
9. At 14 weeks, what did your child receive, multiple choices
10. Polio b. DPT/Hep B/ Hep1 (5 in 1) c. Rotavirus
11. Pneumococcal vaccine e. None, why………………………………
12. At 6 months, what did your child receive, multiple choices
13. Polio b. Measles c. Vitamin
14. I don’t know e. None, why…………………………………….
15. At 9 months, what did your child receive, multiple choices
16. Measles b. Yellow fever c. Measles/Yellow fever
17. None, why ………………………………….
18. At 12 months, did your child receive Vitamin A?
19. Yes b. I don’t know

c. No, why ……………………………………

1. At 18 months, did your child receive Vitamin A and Measles 2?
2. Yes b. I don’t know

c. No, why ……………………………………

1. How many times have your child received Vitamin A after 24 months and above? …………………………………….
