# Supplementary material for: Socio-demographic determinants of childhood immunization incompletion in Koforidua, Ghana
Source: BMC Res Notes. 2018 Sep 10;11:656. doi: 10.1186/s13104-018-3767-x (PMC6131842; doi:10.1186/s13104-018-3767-x)
Supplement: Supplementary file 2 — Additional file 2: Table S1. Immunization schedules before Six (6) months. [file 13104_2018_3767_MOESM2_ESM.docx]

**Additional file 2: Table S1**

**Immunization schedules before Six (6) months**

| **Variables** | **Frequency** | **Percentages** |
| --- | --- | --- |
| **What was your child given at birth** | | |
| BCG/ OPV | 39 | 55.7 |
| BCG | 18 | 25.7 |
| OPV | 7 | 10 |
| I don’t know | 6 | 8.6 |
| **Was your child given polio, DPT/Hep B/Hep 1(5 in 1), Pneumococcal and rotavirus** | | |
| Yes | 64 | 91.4 |
| I don’t know | 6 | 8.6 |
| **What did your child receive at 10 weeks** | | |
| Polio | 3 | 4.3 |
| DPT/ Hep B/ Hep 1 (5 in 1) | 45 | 64.3 |
| Rotavirus | 11 | 15.7 |
| Pneumococcal vaccine | 8 | 11.4 |
| None | 3 | 4.3 |
| **What did your child receive at 14 weeks** | | |
| Polio | 2 | 2.9 |
| DPT/ Hep B/ Hep (5 in 1) | 33 | 47.1 |
| Rotavirus | 9 | 12.9 |
| Pneumococcal vaccine | 20 | 28.6 |
| None | 6 | 8.5 |
