# Supplementary material for: Socio-demographic determinants of childhood immunization incompletion in Koforidua, Ghana
Source: BMC Res Notes. 2018 Sep 10;11:656. doi: 10.1186/s13104-018-3767-x (PMC6131842; doi:10.1186/s13104-018-3767-x)
Supplement: Supplementary file 3 — Additional file 3: Table S2. Immunization schedule after Six (6) months. [file 13104_2018_3767_MOESM3_ESM.docx]

**Additional file 3: Table S2**

Immunization schedule after Six (6) months

| **Variables** | **Frequency** | **Percentages** |
| --- | --- | --- |
| **What was your child given at 6 months?** | | |
| Polio | 9 | 12.9 |
| Measles | 9 | 12.9 |
| Vitamin | 44 | 62.9 |
| I don’t know | 8 | 11.3 |
| **What was your child given at 9 months?** | | |
| Measles | 11 | 15.7 |
| Yellow Fever | 20 | 28.6 |
| Measles/ Yellow fever | 31 | 44.3 |
| None | 8 | 11.4 |
| **Did your child receive Vitamin A at 12 months?** | | |
| Yes | 51 | 72.9 |
| I don’t know | 12 | 17.1 |
| No | 7 | 10 |
| **Did your child receive Vitamin A and Measles 2 at 18 months?** | | |
| Yes | 48 | 68.6 |
| I don’t know | 22 | 31.4 |
| **How many times did your child receive Vitamin A after 24 months?** | | |
| 1 | 11 | 15.7 |
| 2 | 15 | 21.4 |
| 3 | 19 | 27.1 |
| 4 | 4 | 5.7 |
| I don’t know | 21 | 30.1 |
